# Paracetamol, its metabolites, and their transfer between maternal circulation and fetal brain in mono- and combination therapies

**DOI:** 10.1007/s43440-024-00682-6

**Published:** 2025-01-24

**Authors:** Yifan Huang, Fiona Qiu, Katarzyna M. Dziegielewska, Mark D. Habgood, Norman R. Saunders

**Affiliations:** Department of Neuroscience, School of Translational Medicine, Monash University, Melbourne, VIC 3004 Australia

**Keywords:** Paracetamol (acetaminophen), Metabolism, Pregnancy, Drug-drug interaction, Brain entry, Placental transfer

## Abstract

**Background:**

Due to its availability and perceived safety, paracetamol is recommended even during pregnancy and for neonates. It is used frequently alone or in combination with other drugs required for the treatment of various chronic conditions. The aim of this study was to investigate potential effects of drug interactions on paracetamol metabolism and its placental transfer and entry into the developing brain.

**Methods:**

Sprague Dawley rats at postnatal day P4, pregnant embryonic day E19 dams, and non-pregnant adult females were administered paracetamol (15 mg/kg) either as monotherapy or in combination with one of seven other drugs: cimetidine, digoxin, fluvoxamine, lamotrigine, lithium, olanzapine, valproate. Concentrations of parent paracetamol and its metabolites (paracetamol-glucuronide, paracetamol-glutathione, and paracetamol-sulfate) in plasma, cerebrospinal fluid (CSF) and brain were measured by liquid chromatography coupled with tandem mass spectrometry (LC-MS/MS) and their entry into the brain, CSF and transfer across the placenta were estimated.

**Results:**

In monotherapy, concentration of parent paracetamol in plasma, CSF, and brain remained similar and at all ages brain entry was unrestricted. In combination therapies, CSF entry of paracetamol increased following co-treatment with olanzapine. Placental transfer of parent paracetamol remained unchanged, however, transfer of paracetamol-sulfate increased with lamotrigine co-administration. Acutely administered paracetamol was more extensively metabolized in adults compared to younger ages resulting in increased concentration of its metabolites with age.

**Conclusions:**

Developmental changes in the apparent brain and CSF entry of paracetamol appear to be determined more by its metabolism, rather than by cellular control of its transfer across brain and placental barriers.

**Supplementary Information:**

The online version contains supplementary material available at 10.1007/s43440-024-00682-6.

## Introduction

Paracetamol (acetaminophen) is an analgesic and antipyretic medication widely available over the counter in many countries. It is advised as safe for use in neonates and during pregnancy [1, 2], however, in recent years its safety has been questioned [3–5]. It is routinely taken both as a single agent alone or in combination with other medications. However, due to the nature of combination therapy and polypharmacy, potential drug interactions have rarely been considered, and of those described, many are anecdotal case reports with many conflicting results [6]. Even less is known about any possible drug interactions if taken during development or in the early postnatal period and very few published studies investigated in vivo entry of paracetamol into the developing brain or its transfer across the placenta.

Previously we reported experiments that estimated entry of paracetamol into the brain and cerebrospinal fluid (CSF) at different ages in the rat [7]. Results showed a developmental change in the apparent entry of paracetamol with more of the drug reaching the developing than the adult brain. The explanation provided for the observed results was the possibility of progressive maturation of efflux transporters which would more effectively keep the drug from entering the tissue in adults. However, in that study [7], paracetamol concentration was estimated only using its radiolabelled (^3^H) form, leaving open the possibility of potential effects of drug metabolism on the observed paracetamol brain and CSF entry. The main metabolites of paracetamol (paracetamol-glucuronide, paracetamol-glutathione, and paracetamol-sulfate) are more hydrophilic as opposed to the relatively lipophilic parent paracetamol (logP = 0.46, pKa = 9.5) [8], making their movement across brain barriers highly restricted by tight junctions present in these interfaces [9, 10]. Therefore, the aim of the present study was to use liquid chromatography coupled with tandem mass spectrometry (LC-MS/MS) to measure both, parent paracetamol and its metabolites to investigate if rather than maturation of efflux transporters, the observed developmental decline in the drug’s brain entry could be explained by the increase of its metabolism by the more mature liver.

## Methods

### Animals

The animals, the Sprague-Dawley strain of *Rattus norvegicus*, were supplied by the University of Melbourne Biological Research Facility. Adults were housed in groups of 2–4 and litters were housed together in 25 cm x 35 cm x 25 cm cages on Breeders Choice paper bedding with *ad libitum* access to food (fixed formulation dry pellets for rats, Speciality Feeds, Western Australia) and water. The use of animals in this study was approved by the University of Melbourne Ethics Committee (Ethics Approval AEC: 10092) and all animal experimentations were conducted in compliance with the Australian National Health and Research. All animals were handled only by trained experimenters or experienced Animal House staff and every effort was made to minimize the distress and suffering of animals. This study conforms to the ARRIVE guidelines 2.0.

Developmental ages used were dams and fetuses from time-mated females (all primigravida) at embryonic day (E)19, pups at postnatal day (P)4, and 7–8 week-old non-pregnant females. The day a vaginal plug was identified is E0 and P0 is the day of birth. The experimental design was similar to previous studies allowing results to be compared directly with previously published data [7, 11–13].

### Drugs

Equivalent drug doses were selected based on those used in clinical practice [1] (Australian Medicine’s Handbook, 2024) and adjusted to the body weight of the animal as previously described [7, 13] (Table 1).

**Table 1:** List of drugs used, their abbreviation, solvent, dose, supplier and catalogue number. Saline is 0.9% sterile sodium chloride solution. Digoxin and lamotrigine were dissolved in ethanol before dilution in sterile 0.9% sodium chloride solution, the final ethanol concentration was less than a third of the total injectate

Drug	Abbreviation	Solvent	Dose	Supplier	Catalogue #
Paracetamol	PARA	Saline	15 mg/kg	Sigma Aldrich	A7085
Cimetidine	CIM	Saline	11 mg/kg	Sigma Aldrich	C3422
Digoxin	DIG	Ethanol + saline	0.03 mg/kg	Sigma Aldrich	D6003
Fluvoxamine	FLX	Saline	1.5 mg/kg	Sigma Aldrich	F2802
Lamotrigine	LTG	Ethanol + saline	6 mg/kg	Sigma Aldrich	PHR1392
Lithium	Li	Saline	3.2 mg/kg	Sigma Aldrich	310,468
Olanzapine	OLZ	Ethanol	0.15 mg/kg	Sigma Aldrich	PHR1825
Valproate	VPA	Saline	30 mg/kg	Sigma Aldrich	P4543

### Experimental procedure

**Fig. 1:**
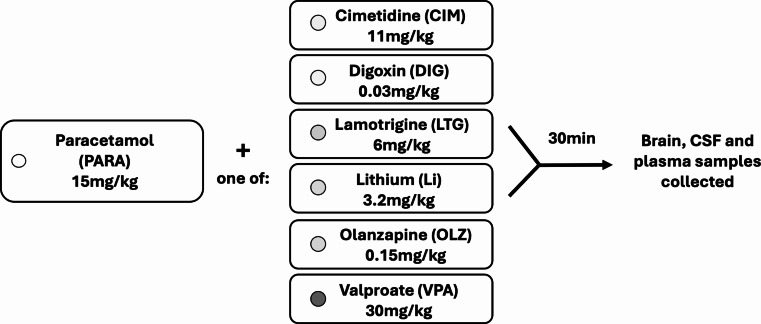
Drug injection protocol. Paracetamol (PARA, 15 mg/kg) was administered to Sprague Dawley rats either alone or in combination with either cimetidine (CIM), digoxin (DIG), lamotrigine (LTG), lithium (Li), olanzapine (OLZ) or valproate (VPA). Brain, CSF (cerebrospinal fluid) and plasma samples were collected 30 min after *ip* (P4 and adult) or *iv* (E19) injection

Concentrations of the parent drug compound, paracetamol, and its metabolites were determined by LC-MS/MS (liquid chromatography coupled with mass spectrometry) in samples of blood plasma, cerebrospinal fluid (CSF), and brain cortex in rats administered the drug as monotherapy or in combination with one of seven other compounds (Table 1). Drug injection protocol is illustrated in Fig. 1.

### Postnatal animals

Male and female postnatal P4 pups and 7–8 week-old non-pregnant females were used. For monotherapy, a single dose of 15 mg/kg paracetamol was administered by intraperitoneal (*ip*) injection. In combination therapy experiments, this dose was accompanied by one of the 7 other drugs (Table 1). For all experiments, animals were terminally anesthetized 30 min after the drug injection using inhaled isoflurane (IsoFlo 100% w/w, Abbott Laboratories), and blood, CSF, and brain samples were collected. Blood was collected directly from the right cardiac ventricle, CSF from the *cisterna magna*, and cortical brain samples from the frontal/parietal lobes dorsal to the lateral ventricles as described previously [7, 12–15].

### Pregnant dams and fetal E19 animals

Pregnant E19 dams were anesthetized with *ip* urethane injection (25% w/v urethane, Sigma, 1 ml/100 g body weight) and placed on a 39 ºC heating pad in the supine position. An endotracheal catheter was inserted to maintain a clear airway. Catheters were inserted into the femoral vein and artery for *iv* drug administration and arterial blood sampling respectively. The cannula was flushed with 0.5 ml of heparinized saline (Hospira Inc, 5,000 units/ml) after drug administration and following each arterial blood sample. Following drug administration to the dams, fetuses were exteriorized and serially collected starting at 30 min post-maternal injection. All fetuses were collected within 120 min of the maternal injection. At collection, the viability of each fetus was assessed (presence of a heartbeat, the colour of the umbilical vessels) and blood, CSF, and brain samples were taken along with a time-matched maternal blood sample from the femoral artery. At the end of the experiment, a final maternal blood sample was taken directly from the left cardiac ventricle, and CSF and brain samples were collected [7, 13, 15, 16].

### Liquid chromatography coupled with tandem mass spectrometry (LC-MS/MS)

This method has been described previously for valproate [12], lamotrigine [17, 18], and olanzapine [13] and was applied in the present study. Simultaneous quantification was performed for all compounds in plasma, brain, and CSF samples following the addition of respective stable isotope-labeled internal standards. Compound preparation and specifications are listed in Tables 2 and 3. Paracetamol refers to the parent drug compound only.

**Table 2:** List of compounds used in LC-MS/MS (liquid chromatography coupled with tandem mass spectrometry) analysis. The supplier and catalogue number are detailed along with solvent, stock and internal standard concentrations

Compound name	Supplier	Catalogue no.	Solvent	Stock Concentration	Internal standard concentration
Paracetamol-13C6	Chembridge Isotope	CLM-10,619-PK	Methanol	1 mg/ml	10 µg/ml
Paracetamol-glutathione-d3	Toronto Research Chemicals	A161226	Methanol	0.2 mg/ml	0.1 µg/ml
Paracetamol-glucuronide-d3	Toronto Research Chemicals	A158502	Methanol	2 mg/ml	1 µg/ml
Paracetamol-sulfate-d3	Toronto Research Chemicals	A161232	Methanol: water 2:1	0.666 mg/ml	1 µg/ml

**Table 3:** Normalized collision energies (NCE), monitored product ion m/z and retention time of unlabelled and labelled Paracetamol and its metabolites (paracetamol-glutathione, Paracetamol-Glucuronide and Paracetamol sulfate). Peak areas in extracted ion chromatogram of monitored product ions were extracted for quantitative analysis of drugs in each sample using Skyline 21.2 (RRID: SCR_014080)

Compound name	Normalized collision energies (NCE)	MS/MS transition	Retention time (min)
Paracetamol	31%	110.0593 to 152.0711	3.2
Paracetamol-glutathione	19%	328.0952 to 457.1393	3.2
Paracetamol-glucuronide	16%	113.0177 to 326.0875	5.5
Paracetamol-sulfate	28%	152.0705 to 232.0279	2.8
Paracetamol-13C6	31%	116.0795 to 158.0913	3.2
Paracetamol-glutathione-d3	19%	331.1145 to 460.1581	3.2
Paracetamol-glucuronide-d3	16%	113.0177 to 329.1065	5.5
Paracetamol-sulfate-d3	28%	155.0889 to 235.0468	2.8

Linear response range was determined in untreated control rat plasma for each compound at 0.1, 1, 10, 50, 100, 200, 400, and 800 µg/ml. The ranges of linear responses were 0.1–200 µg/ml for paracetamol-13 C, 0.1–100 µg/ml for paracetamol-glutathione-d3, 0.1–200 µg/ml for paracetamol-glucuronide-d3 and 0.1–200 µg/ml for paracetamol-sulfate-d3 (Supplementary Figure S1). Paracetamol-glutathione was below the linear response range in all of the experimental samples measured and therefore not reported in this study.

### Brain or CSF entry and placental transfer

Drug entry in this study refers to the accumulative amount of the drug that entered the brain or the CSF from blood in 30 min following injections [14]. To estimate the entry of paracetamol and its metabolites into the brain or CSF Eq. 1 was used

1 $$\eqalign{& Brain\ or\,CSF\,entry = \cr & \,\,\,\,\,\,\,\,{\matrix{Concentration\,of\,compound \hfill \cr \,\,\,\,\,\,\,\,in\,Brain\ or\,CSF(\mu g/ml) \hfill \cr} \over \matrix{Concentration\,of\,compound \hfill \cr \,\,\,\,\,\,\,\,\,\,\,\,\,\,\,\,\,\,\,in\,plasma(\mu g/ml) \hfill \cr} } \times 100\% \cr} $$

To estimate placental transfer Eq. 2 was used:

2 $$\eqalign{& Placental\,transfer = \cr & {\matrix{Compound\,concentration\,in \hfill \cr \,\,\,fetal\,plasma(\mu g/ml)at\,time\,x \hfill \cr} \over \matrix{Average\,compound\,concentration\,in\, \hfill \cr \,\,maternal\,plasma(\mu g/ml)up\,to\,time\,x \hfill \cr} } \times 100\% \cr} $$

$$\:x=fetal\:sampling\:time$$

### Statistical analysis

An F test was used to test for equal variance between the groups and Shapiro-Wilk test was used to test normality due to small group sizes. As some results did not have normal distribution or equal variance, the differences between all comparison groups were also analyzed using the Kruskal-Wallis test with Dunn’s post hoc test for multiple comparisons. *p* ≤ 0.05 was considered statistically significant. All results displayed using non-parametric tests and data from all experiments are expressed as box plots. The boxes represent the median and interquartile (IQR: first quartile – third quartile) range, the whiskers represent the minimum- maximum values. All statistical analyses were performed using Graphpad Prism 10.

## Results

### Brain and CSF entry of paracetamol and its metabolites in monotherapy

**Fig. 2:**
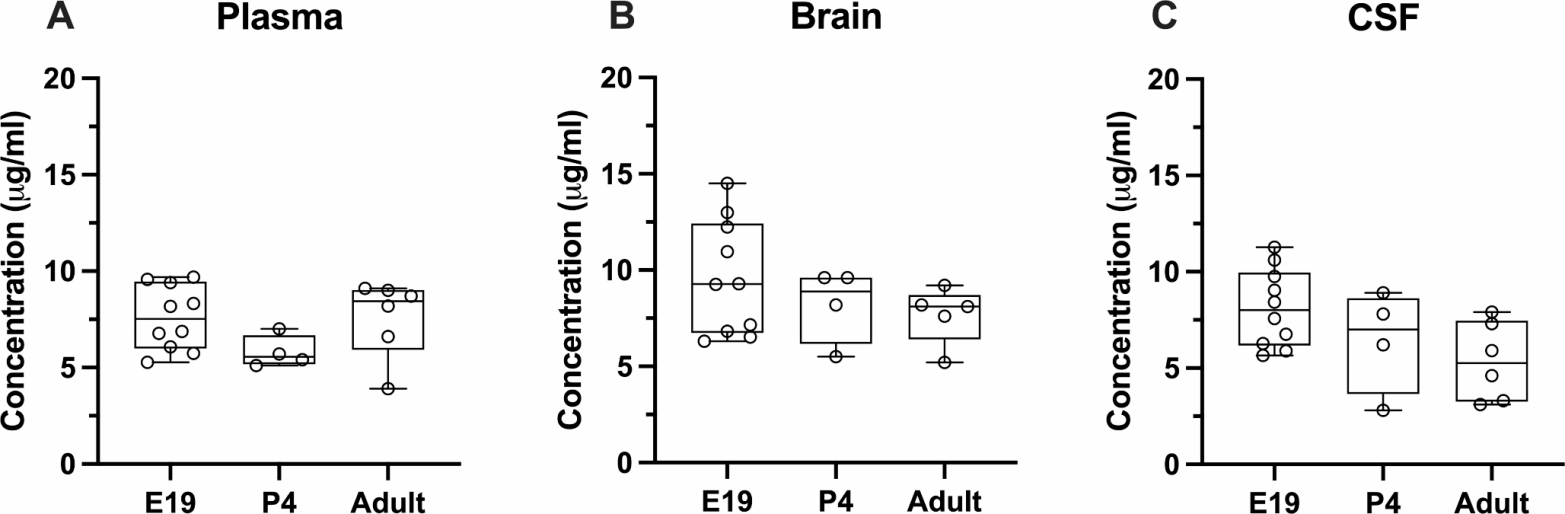
Concentration of paracetamol (µg/ml) in (**A**) plasma, (**B**) brain cortex and (**C**) CSF (cerebrospinal fluid) 30 min after a single dose (15 mg/kg) at E19 (embryonic day 19; *iv*), P4 (postnatal day 4; *ip*) and adult (*ip*). Note each point represents an individual animal. Differences between groups were analyzed using a Kruskal-Wallis test followed by Dunn’s post hoc test. Boxes represent the median and first quartile (Q1)- third quartile (Q3) range, whiskers represent the minimum- maximum value

The concentration of paracetamol in plasma, brain cortex, and CSF was measured using LC-MS/MS 30 min after the *ip* drug administration at three ages: E19, P4, and adult. As shown in Fig. 2, brain and CSF concentrations remained at around 5–10 µg/ml at all ages.

**Fig. 3:**
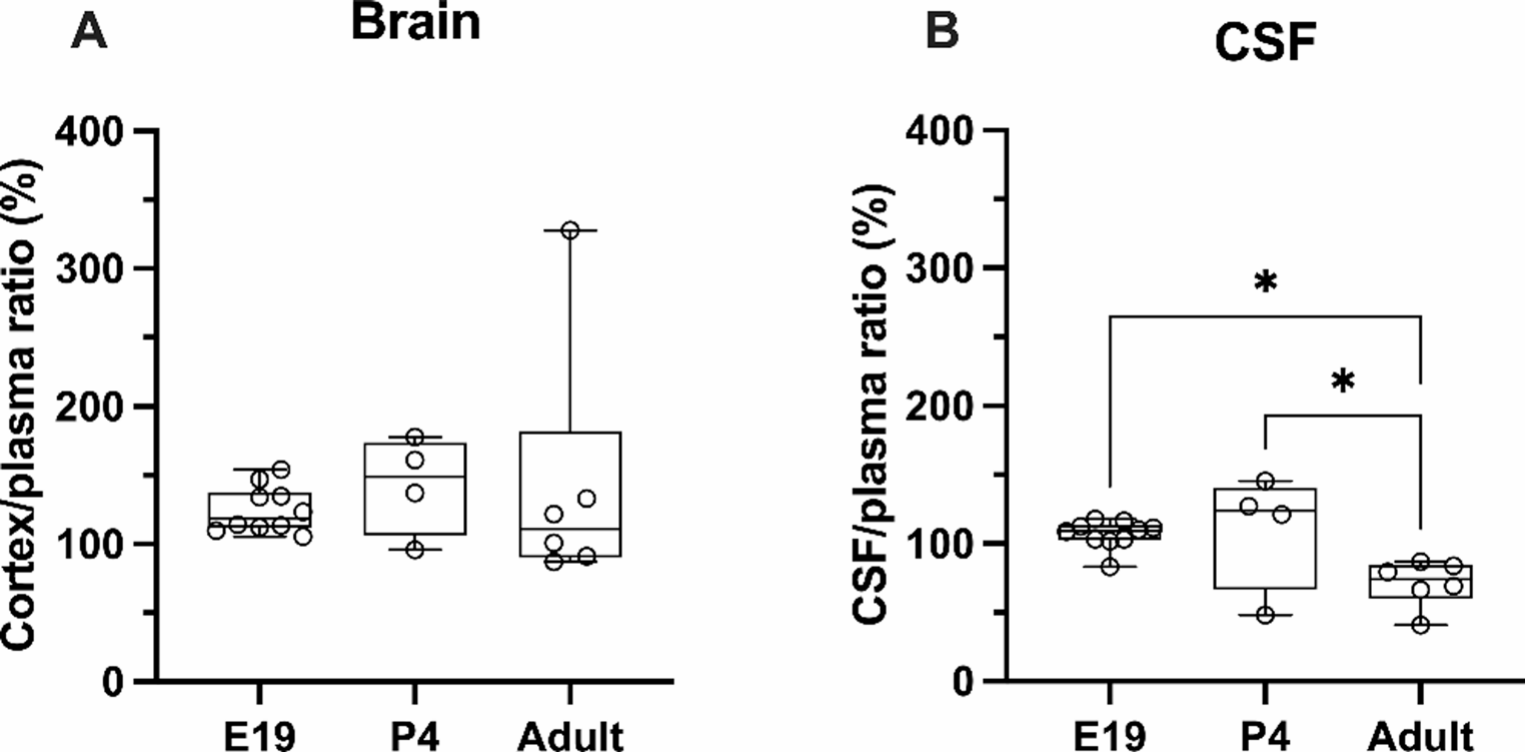
Paracetamol concentration ratios (%): (**A**) brain cortex/plasma, (**B**) CSF (cerebrospinal fluid)/plasma ratios 30 min after a single dose (15 mg/kg) at E19 (embryonic day 19; *iv*), P4 (postnatal day 4; *ip*) and adult (*ip*). Note each point represents an individual animal. Differences between groups were analyzed using a Kruskal-Wallis test followed by Dunn’s post hoc test. Boxes represent the median and first quartile (Q1)- third quartile (Q3) range, whiskers represent the minimum- maximum value. *p < 0.05

Consequently, the entry of paracetamol into the brain, calculated as a ratio (brain/plasma concentration ratio, Eq. 1), was also similar at just over 100% at all ages (Fig. 3A). Entry of paracetamol into the CSF was also around 100% at E19 and P4, however, it was significantly decreased in the adult (Kruskal–Wallis test, H = 8.83, N_1_ = 10, N_2_ = 4, N_3_ = 6; *p* = 0.0373 and *p* = 0.0248 compared to E19 and P4 respectively, Fig. 3B), most likely due to a small decrease in the drug’s CSF concentration (Fig. 2C). Taken together these results demonstrated that paracetamol’s entry into the brain was not restricted and there was a relatively free exchange of the drug between all 3 compartments (blood plasma, brain, and CSF).

### Concentration of paracetamol and its metabolites in plasma following combination therapy

**Fig. 4:**
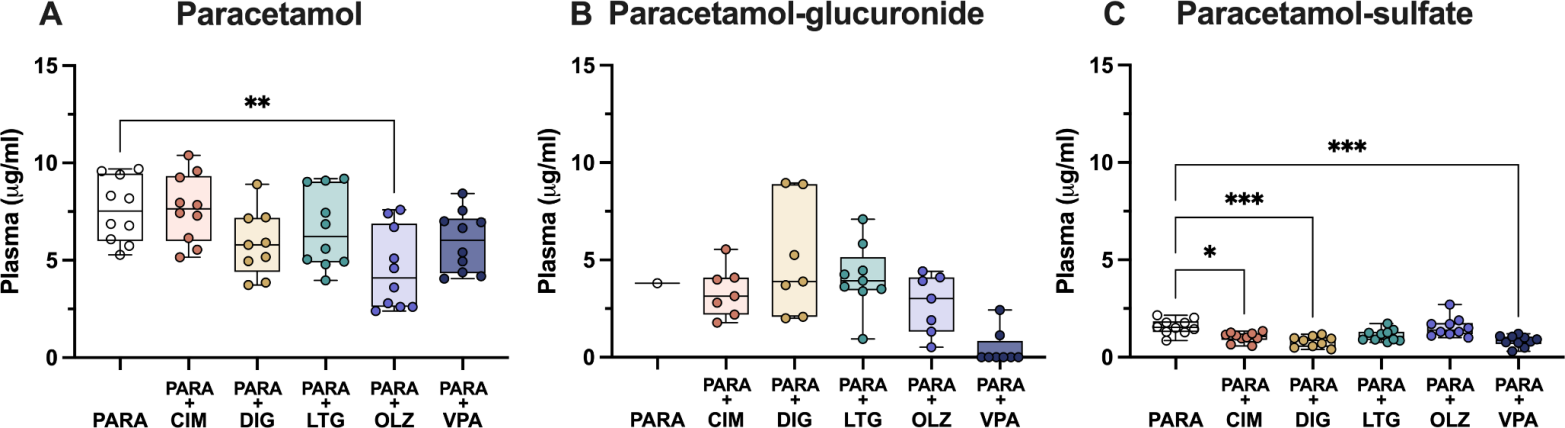
Concentration (µg/ml) at E19 (embryonic day 19) of (**A**) paracetamol, (**B**) paracetamol-glucuronide and (**C**) paracetamol-sulfate present in plasma 30 min after a single maternal *iv* injection of paracetamol (15 mg/kg) either as monotherapy or in combination with: cimetidine (CIM, 11 mg/kg), digoxin (DIG, 0.03 mg/kg), lamotrigine (LTG, 6 mg/kg), olanzapine (OLZ, 0.15 mg/kg) or valproate (VPA, 30 mg/kg). Note each point represents an individual animal. Differences between groups were analyzed using a Kruskal-Wallis test followed by Dunn’s post hoc test. Boxes represent the median and first quartile (Q1)- third quartile (Q3) range, whiskers represent the minimum- maximum value. *p < 0.05, **p < 0.01, ***p < 0.001

In order to investigate if co-administration of paracetamol with another drug could affect its metabolism at different ages, the concentration of parent paracetamol as well as its metabolites was estimated in blood plasma. At E19, the plasma concentration of paracetamol in monotherapy was a median of 7.5 µg/ml (IQR = 2.9, Fig. 4A). In combination therapies, the only statistically significant difference was detected following co-administration with olanzapine, where the concentration of paracetamol decreased significantly to 4.1 µg/ml (IQR = 3.7; Kruskal–Wallis test, H = 15.8, N_1_ = 10, N_2_ = 10, N_3_ = 9, N_4_ = 10, N_5_ = 10, N_6_ = 10, *p* = 0.007). Concentration of paracetamol-glucuronide appeared to be lower following valproate co-administration compared to paracetamol monotherapy, however due to the limited sample number statistical differences could not be calculated (Fig. 4B). The biggest changes were detected for paracetamol-sulfate. In comparison to its concentration in monotherapy (1.5 µg/ml, IQR = 0.4; Fig. 4C), paracetamol concentration was significantly lower following co-administration with cimetidine (reduced to 1.1 µg/ml, IQR = 0.2), digoxin (0.9, IQR 0.4 µg/ml) and valproate (0.9, IQR = 0.3 µg/ml; Kruskal–Wallis test, H = 28.98, N_1_ = 10, N_2_ = 10, N_3_ = 9, N_4_ = 10, N_5_ = 10, N_6_ = 10, *p* = 0.045, *p* = 0.0003 and *p* = 0.0005 respectively). Results are illustrated in Fig. 4C.

**Fig. 5:**
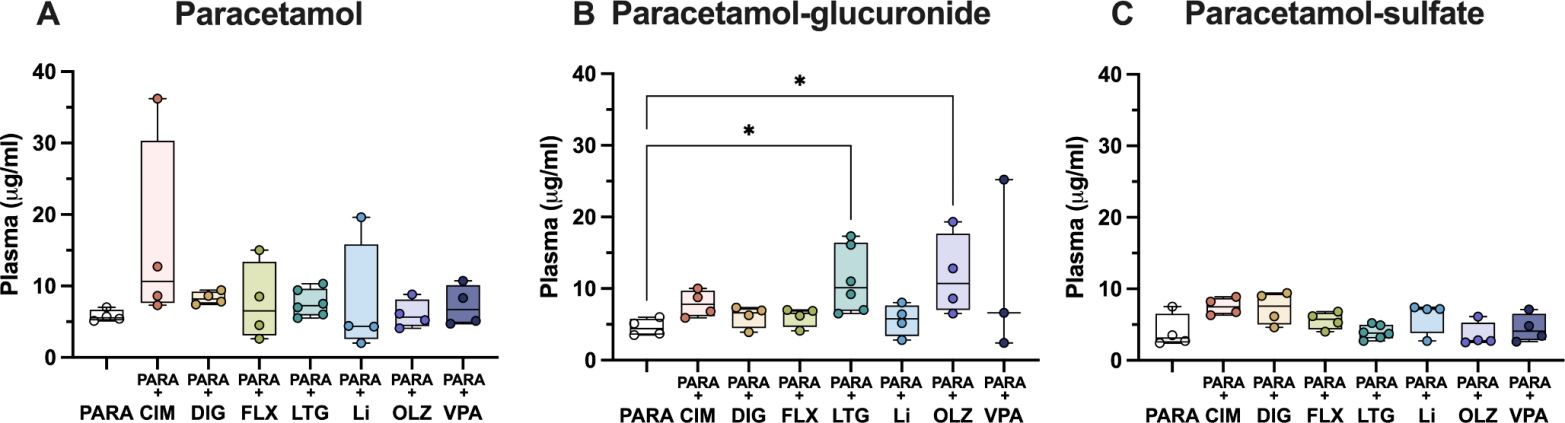
Concentration (µg/ml) at P4 (postnatal day 4) of (**A**) paracetamol, (**B**) paracetamol-glucuronide and (**C**) paracetamol-sulfate present in plasma 30 min after a single *ip* injection of paracetamol (15 mg/kg) either as monotherapy or in combination with: cimetidine (CIM, 11 mg/kg), digoxin (DIG, 0.03 mg/kg), fluvoxamine (FLX, 1.5 mg/kg), lamotrigine (LTG, 6 mg/kg), lithium (Li, 3.2 mg/kg), olanzapine (OLZ, 0.15 mg/kg) or valproate (VPA, 30 mg/kg). Note each point represents an individual animal. Differences between groups were analyzed using a Kruskal-Wallis test followed by Dunn’s post hoc test. Boxes represent the median and first quartile (Q1)- third quartile (Q3) range, whiskers represent the minimum- maximum value. *p < 0.05

At P4, the concentration of paracetamol and paracetamol-sulfate in plasma did not change following combination therapy with any of the drugs investigated remaining at around 5 µg/ml (Kruskal–Wallis test, paracetamol: H = 8.834, N_1_ = 4, N_2_ = 4, N_3_ = 4, N_4_ = 4, N_5_ = 4, N_6_ = 6, N_7_ = 4, N_8_ = 4, *p* = 0.264, 0.887, > 1, >1, > 1, >1, > 1; paracetamol-sulfate: H = 14.76, N_1_ = 4, N_2_ = 4, N_3_ = 4, N_4_ = 4, N_5_ = 4, N_6_ = 6, N_7_ = 4, N_8_ = 4, *p* = 0.177, 0.327, > 1, 0.827, > 1, >1, > 1). On the other hand, the concentration of paracetamol-glucuronide (4.4 µg/ml, IQR = 1.7) was significantly higher after co-administration with lamotrigine (10.1 µg/ml, IQR = 8.0) and olanzapine (10.7 µg/ml, IQR = 6.4; Kruskal–Wallis test, H = 14.19, N_1_ = 4, N_2_ = 4, N_3_ = 4, N_4_ = 4, N_5_ = 5, N_6_ = 4, N_7_ = 4, *p* = 0.0163 and 0.0403 respectively), as shown in Fig. 5.

**Fig. 6:**
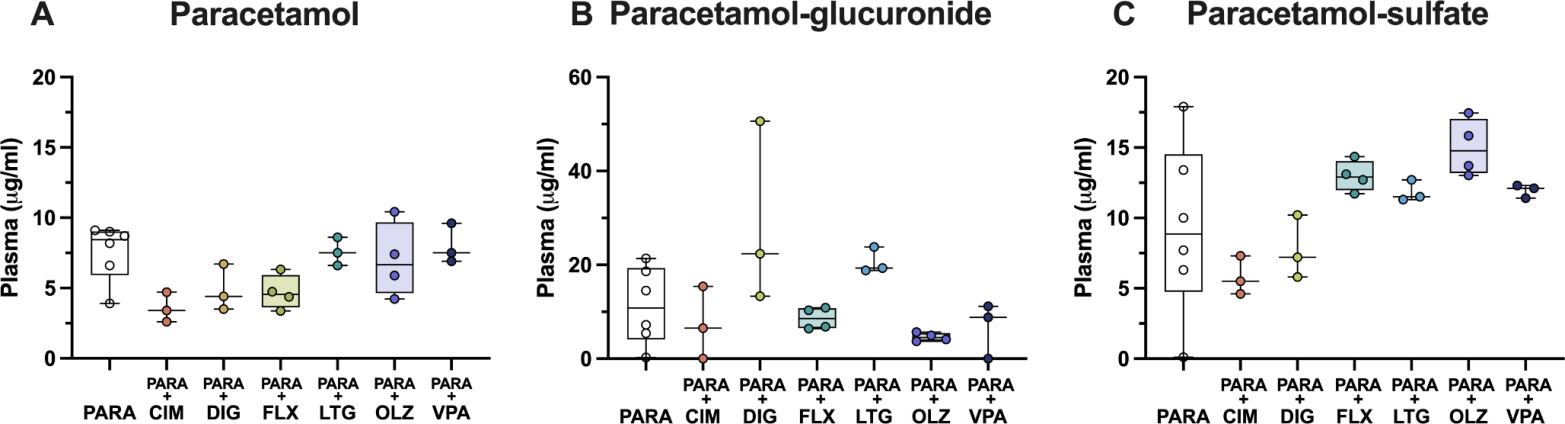
Concentration (µg/ml) in adult of (**A**) paracetamol, (**B**) paracetamol-glucuronide and (**C**) paracetamol-sulfate present in plasma 30 min after a single *ip* injection of paracetamol (15 mg/kg) either as monotherapy or in combination with: cimetidine (CIM, 11 mg/kg), digoxin (DIG, 0.03 mg/kg), fluvoxamine (FLX, 1.5 mg/kg), lamotrigine (LTG, 6 mg/kg), olanzapine (OLZ, 0.15 mg/kg) or valproate (VPA, 30 mg/kg). Note each point represents an individual animal. Differences between groups were analyzed using a Kruskal-Wallis test followed by Dunn’s post hoc test. Boxes represent the median and first quartile (Q1)- third quartile (Q3) range, whiskers represent the minimum- maximum value

In the adults, there were no significant changes observed in parent paracetamol, paracetamol-glucuronide, or paracetamol-sulfate concentration following combination therapy with any of the drugs investigated using Kruskal–Wallis test (Fig. 6, paracetamol: H = 12.72, N_1_ = 6, N_2_ = 3, N_3_ = 3, N_4_ = 4, N_5_ = 3, N_6_ = 4, N_7_ = 3, *p* = 0.0719, 0.595, 0.236, > 1, >1, > 1; paracetamol-glucuronide: H = 13.68, N_1_ = 6, N_2_ = 3, N_3_ = 3, N_4_ = 4, N_5_ = 3, N_6_ = 4, N_7_ = 3, p = > 1, 0.696, > 1, 0.614, 0.609, > 1; paracetamol-sulfate: H = 14.83, N_1_ = 6, N_2_ = 3, N_3_ = 3, N_4_ = 4, N_5_ = 3, N_6_ = 4, N_7_ = 3, *p* = 0.938, > 1, 0.908, > 1, 0.169 and > 1).

### Brain and CSF entry of paracetamol and its metabolites in combination therapy

**Fig. 7:**
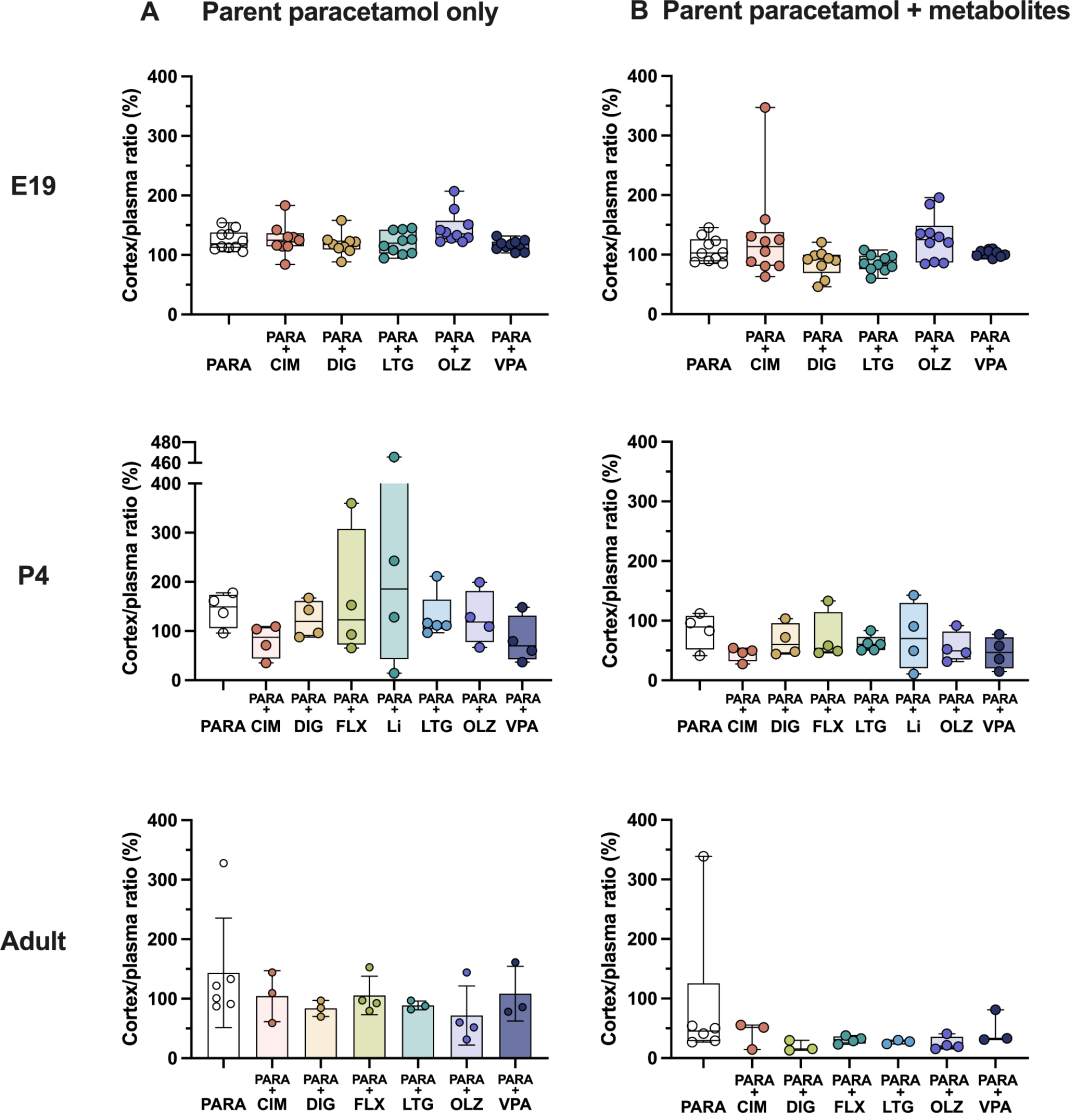
Brain entry of (**A**) paracetamol alone or (**B**) paracetamol with its metabolites at E19 (embryonic day 19), P4 (postnatal day 4) and adult after a single *iv* or *ip* injection of paracetamol (15 mg/kg) either as monotherapy or in combination with: cimetidine (CIM, 11 mg/kg), digoxin (DIG, 0.03 mg/kg), fluvoxamine (FLX, 1.5 mg/kg), lamotrigine (LTG, 6 mg/kg), lithium (Li, 3.2 mg/kg), olanzapine (OLZ, 0.15 mg/kg) or valproate (VPA, 30 mg/kg). Note that data have been re-calculated in µM for comparative purposes. Each point represents an individual animal. Differences between groups were analyzed using a Kruskal-Wallis test followed by Dunn’s post hoc test. Boxes represent the median and first quartile (Q1)- third quartile (Q3) range, whiskers represent the minimum- maximum value. *p < 0.05

**Fig. 8:**
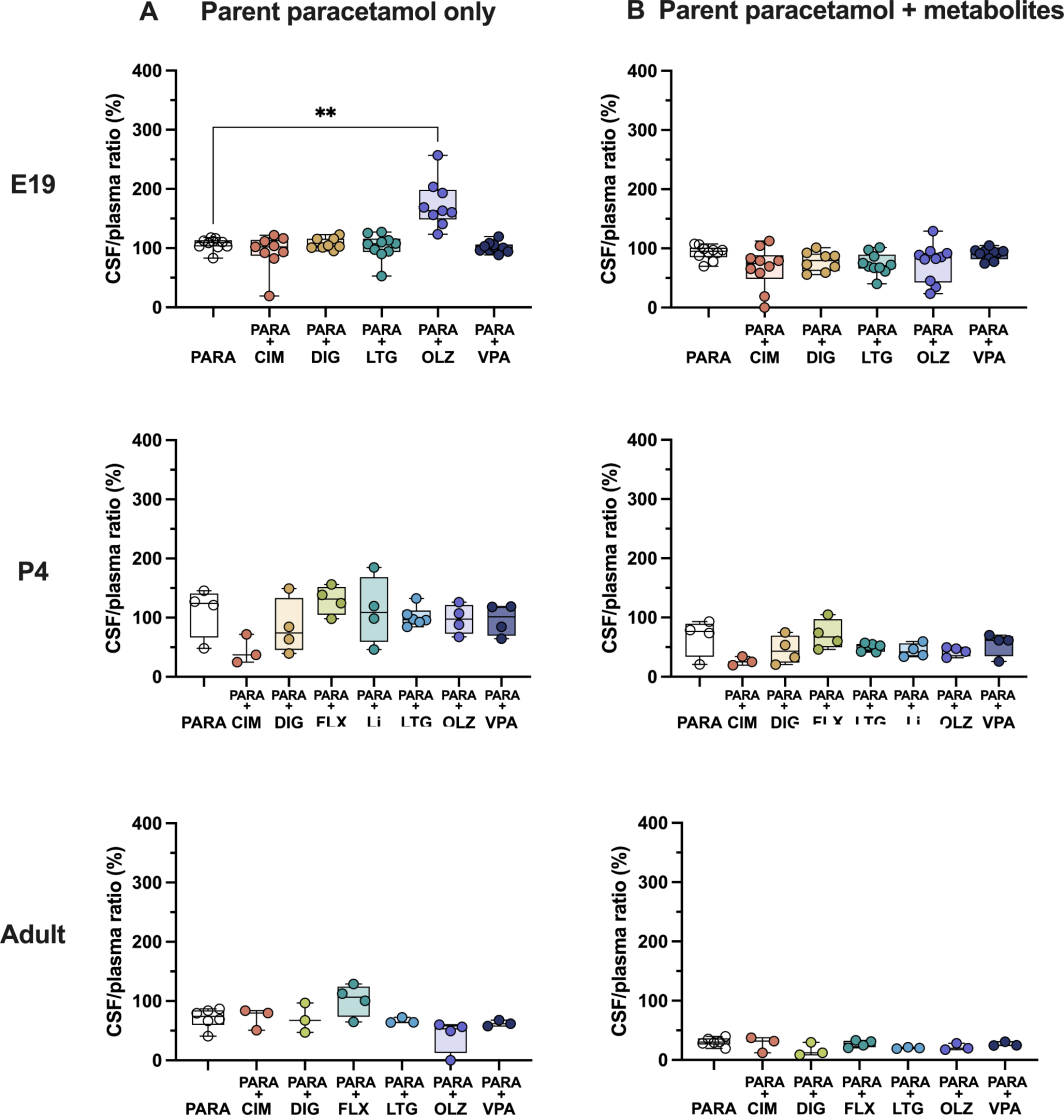
CSF (cerebrospinal fluid) entry of (**A**) paracetamol alone or (**B**) paracetamol with its metabolites at E19 (embryonic day 19), P4 (postnatal day 4) and adult after a single *iv* or *ip* injection of paracetamol (15 mg/kg) either as monotherapy or in combination with: cimetidine (CIM, 11 mg/kg), digoxin (DIG, 0.03 mg/kg), fluvoxamine (FLX, 1.5 mg/kg), lamotrigine (LTG, 6 mg/kg), lithium (Li, 3.2 mg/kg), olanzapine (OLZ, 0.15 mg/kg) or valproate (VPA, 30 mg/kg). Note that data have been re-calculated in µM for comparative purposes. Each point represents an individual animal. Differences between groups were analyzed using a Kruskal-Wallis test followed by Dunn’s post hoc test. Boxes represent the median and first quartile (Q1)- third quartile (Q3) range, whiskers represent the minimum- maximum value. **p < 0.01

Results of experiments at three ages (E19, P4, and adult) measuring brain and CSF entry of paracetamol when administered alone or in combination therapies are illustrated in Figs. 7 and 8. Data are presented using values for concentrations (µM) of either only paracetamol or when the concentration of paracetamol was added to those of its metabolites (paracetamol-glucuronide and paracetamol-sulfate). Entry into the brain and CSF was calculated using Eq. 1. Numerical data used to obtain these values are shown in Supplementary Tables S1-3.

### Brain entry

Brain entry of parent paracetamol was similar at all 3 ages at around 100% as shown above in Fig. 3. However, only at E19 brain entry of paracetamol calculated together with its metabolites remained at around 100% (Fig. 7). In contrast, in P4 animals, entry of paracetamol measured together with its metabolites appeared to be lower at around 50%. In the adults, the brain entry of paracetamol and its metabolites combined was even lower, at around 25%. Possible explanation for this developmental decrease in the apparent brain entry is discussed in the Discussion. None of the drug combination therapies altered brain entry of paracetamol, or paracetamol combined with its metabolites, at any of the ages investigated (Fig. 7).

### CSF entry

CSF entry of paracetamol itself remained relatively similar throughout development at around 75–100% (Fig. 8) and was similar to that for the brain as shown in Fig. 7 above. At E19 the entry remained at around 100% even when paracetamol concentrations were combined with those of its metabolites. At the older ages, these values appeared to decrease to around 40% at P4 and around 20% in the adults (Fig. 8). In combination therapies an increased paracetamol entry into the CSF was observed at E19 following co-administration of olanzapine from 109.5% (IQR = 9.3) in monotherapy to 163.0% (IQR = 43.7; Kruskal–Wallis test, H = 26.03, N_1_ = 10, N_2_ = 9, N_3_ = 8, N_4_ = 10, N_5_ = 9, N_6_ = 10, *p* = 0.0067) as shown in Fig. 8A. This difference was no longer present when paracetamol metabolites were also included (Fig. 8B). At P4 there were no significant differences in paracetamol CSF entry following any of the combination therapies, remaining at around 100% (Fig. 8A). However, when the metabolites were added into the calculation, this value appeared to be lower at around 40% (Fig. 8B). In the adult, CSF entry of paracetamol remained around 70% and no significant differences were found between values obtained in monotherapy or any of the combination therapies (Fig. 8A; Kruskal–Wallis test, parent paracetamol only: H = 10.85, N_1_ = 6, N_2_ = 3, N_3_ = 3, N_4_ = 4, N_5_ = 3, N_6_ = 4, N_7_ = 3, p = > 1, > 1, >1, > 1, 0.192, > 1; parent paracetamol + metabolites: H = 10.14, N_1_ = 6, N_2_ = 3, N_3_ = 3, N_4_ = 4, N_5_ = 3, N_6_ = 3, N_7_ = 3, p = > 1, 0.392, > 1, 0.552, 0.716, > 1), while entry of paracetamol together with its conjugated metabolites was around 20% both in monotherapy and in all combination therapies (Fig. 8B).

### Placental transfer of paracetamol and its metabolites

**Fig. 9:**
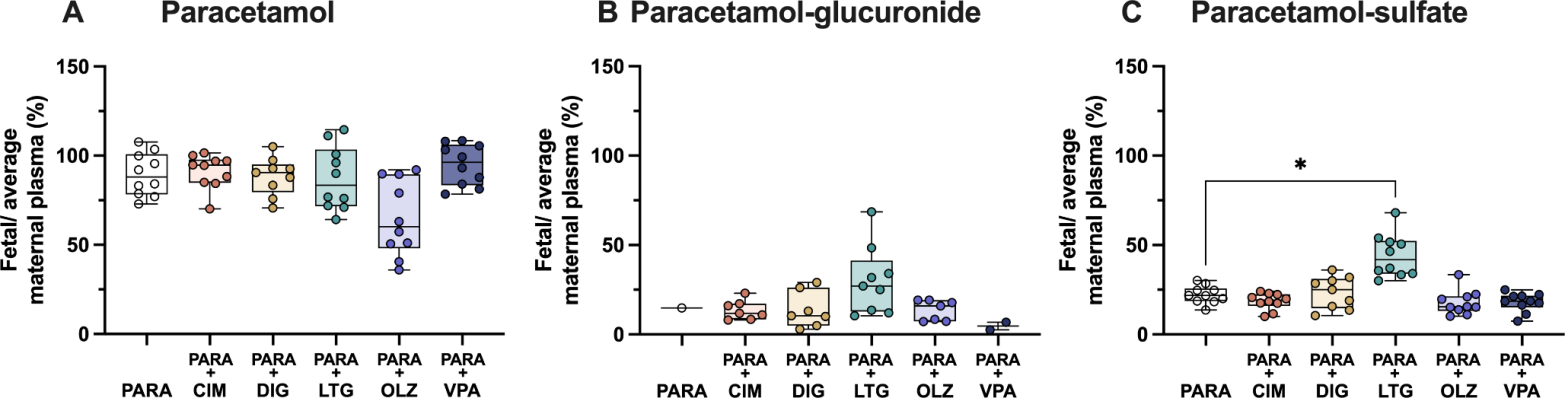
Placental transfer at E19 (embryonic day 19) of (**A**) paracetamol, (**B**) paracetamol-glucuronide and (**C**) paracetamol-sulfate as calculated by fetal/average maternal plasma ratio (%) after a single *iv* injection of paracetamol (15 mg/kg) either as monotherapy or in combination with: cimetidine (CIM, 11 mg/kg), digoxin (DIG, 0.03 mg/kg), lamotrigine (LTG, 6 mg/kg), olanzapine (OLZ, 0.15 mg/kg) or valproate (VPA, 30 mg/kg). Note each point represents an individual animal, paracetamol-glucuronide values below the level of detection (0.1 µg/ml) were not included. All values are available at Supplementary Table S1. Differences between groups were analyzed using a Kruskal-Wallis test followed by Dunn’s post hoc test. Boxes represent the median and first quartile (Q1)- third quartile (Q3) range, whiskers represent the minimum- maximum value. *p < 0.05

Transfer of paracetamol and its metabolites across the placenta from dam to fetus was estimated by comparing levels of the drug in fetal and maternal plasma (Eq. 2) following both monotherapy and combination therapy. In monotherapy, the placental transfer of paracetamol was 89 ± 12% and this was unaltered following combination therapy with any of the drugs investigated (Fig. 9A; Kruskal–Wallis test, H = 12.90, N_1_ = 10, N_2_ = 10, N_3_ = 9, N_4_ = 10, N_5_ = 10, N_6_ = 10, p = > 1, > 1, >1, 0.083, > 1). Paracetamol-glucuronide concentration levels detected were very low in monotherapy and as only a single sample was within the linear response range, no statistical analyses were conducted. However, concentrations of this metabolite could be measured in combination therapy groups resulting in placental transfer ranging from ~ 13–30% (Fig. 9B). Paracetamol-sulfate transfer across the placenta was 21.6% (IQR = 5.7) in monotherapy and the only significant increase detected was with lamotrigine co-therapy (Figs. 9C and 41.8% IQR = 16.9; Kruskal–Wallis test, H = 27.15, N_1_ = 10, N_2_ = 10, N_3_ = 9, N_4_ = 10, N_5_ = 10, N_6_ = 10, *p* = 0.0137).

### Entry of paracetamol into fetal brain and CSF in relation to its concentration in maternal circulation

**Fig. 10:**
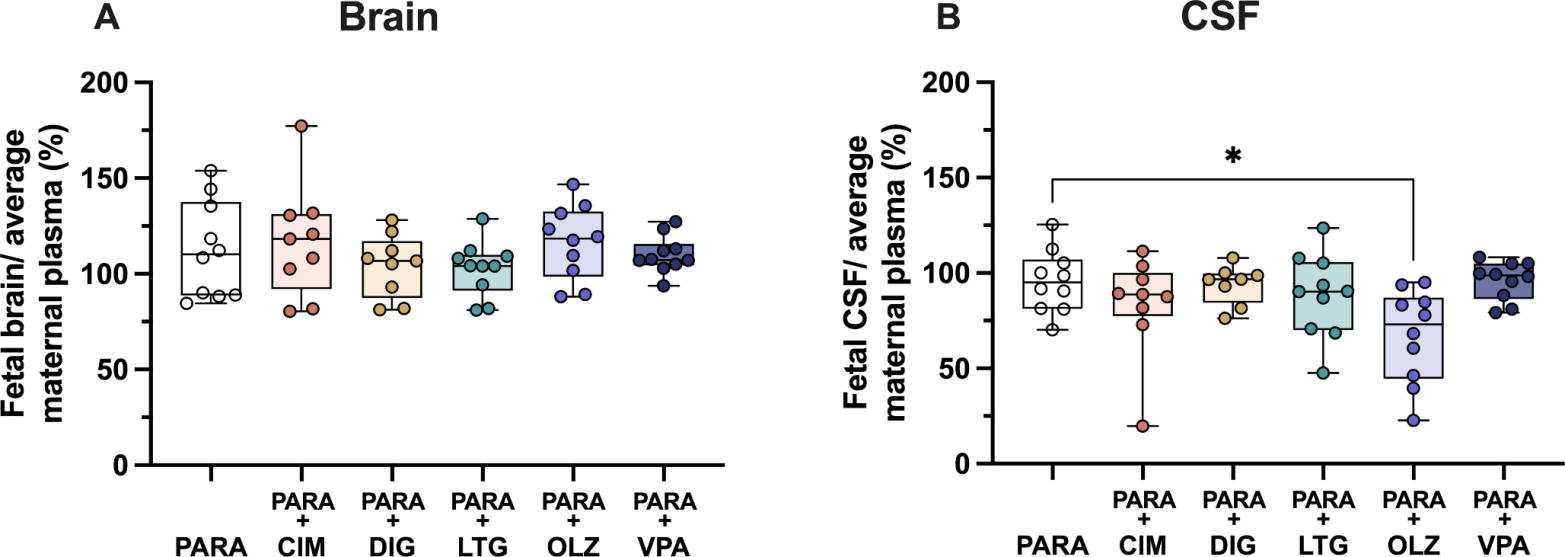
Transfer of paracetamol from the maternal plasma to: (**A**) fetal brain cortex and (**B**) CSF (cerebrospinal fluid) as calculated by fetal brain or CSF/average maternal plasma ratio (%) after a single *iv* injection of paracetamol (15 mg/kg) either as monotherapy or in combination with: cimetidine (CIM, 11 mg/kg), digoxin (DIG, 0.03 mg/kg), lamotrigine (LTG, 6 mg/kg), olanzapine (OLZ, 0.15 mg/kg) or valproate (VPA, 30 mg/kg. Note each point represents an individual animal. All values are available at Supplementary Table S1. Differences between groups were analyzed using a Kruskal-Wallis test followed by Dunn’s post hoc test. Boxes represent the median and first quartile (Q1)- third quartile (Q3) range, whiskers represent the minimum- maximum value. *p < 0.05

Entry of paracetamol into the fetal brain and CSF can also be estimated by comparing directly its concentrations in maternal circulation to that in the fetal brain and CSF in both mono- and combination therapies (Fig. 10). Transfer of paracetamol into the fetal brain remained at just over 100% across all treatment groups, however, compared to paracetamol monotherapy, its transfer into the fetal CSF decreased significantly in co-therapy with olanzapine from 95.1% (IQR = 20.3) to 73.1% (IQR = 36.5; Kruskal–Wallis test, H = 11.74, N_1_ = 10, N_2_ = 9, N_3_ = 8, N_4_ = 10, N_5_ = 10, N_6_ = 10, *p* = 0.0293).

## Discussion

Experiments described in the present study showed that at different stages of development in the rat, metabolism plays an integral role in determining paracetamol entry into the brain and CSF both in monotherapy and when challenged with drug interactions.

**Fig. 11:**
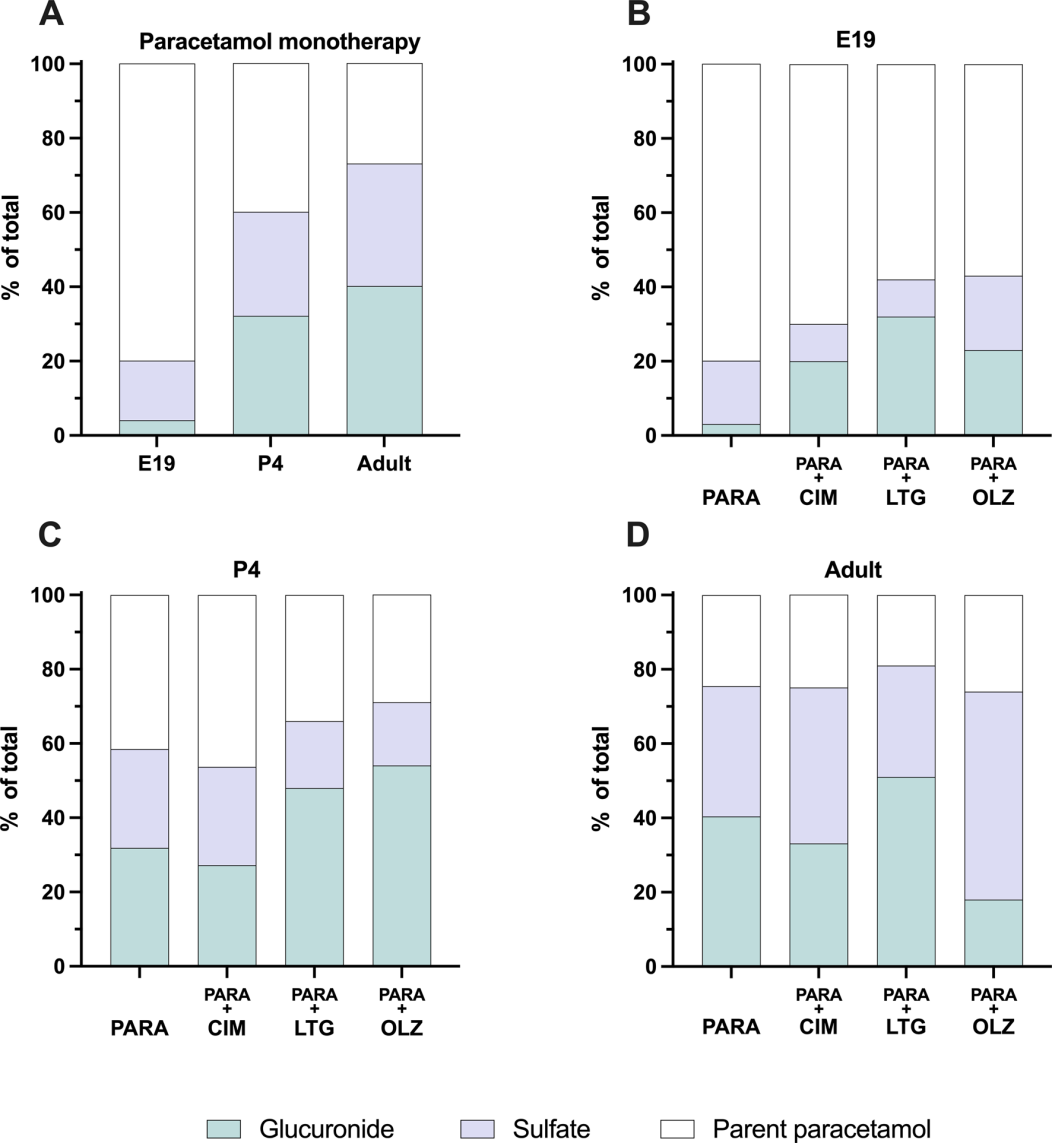
Percentage of paracetamol, paracetamol-glucuronide and paracetamol-sulfate present in plasma at E19 (embryonic day 19), P4 (postnatal day 4) and adult 30 min after a single *iv* or *ip* injection of paracetamol (15 mg/kg). Note concentrations in plasma were converted to µM for this calculation. Values from paracetamol monotherapy animals in Figs. 3, 4 and 5

Previously we have reported results from similar experiments using paracetamol labelled with tritium [7]. However, due to this radiolabelling, this method could not distinguish between the parent drug and its metabolites. The tritiated methyl groups in radioactive [2,6-^3^H] paracetamol are conserved in its metabolites and although its glucuronide conjugate may displace one ^3^H, there would still be at least one radioactive ^3^H remaining. From the present study it can be seen that a higher proportion of administered paracetamol was metabolized in older animals (illustrated schematically in Fig. 11). As conjugated metabolites are highly water soluble, their ability to transfer across brain barriers would be greatly reduced [19–21]. Hence the developmental decline observed could be due to increasing the pool of labelled metabolites in plasma rather than modulation of ABC efflux transporters as previously suggested [7]. Indeed, present results showed that from E19 to adult, concentration of paracetamol itself remained similar but concentrations of its metabolites changed significantly with age. Previous studies in humans also found that after a single dose of the drug (14.4 mg/kg), concentration of paracetamol itself in plasma was similar in preterm infants and adults [22].

To illustrate these relative changes, the concentration of each compound (paracetamol, paracetamol-glucuronide and paracetamol-sulfate) was presented as a percentage of the total (Fig. 11). At E19, only a small proportion of paracetamol was metabolized into its glucuronide (2%) and sulfate (12%) conjugates with 86% of the paracetamol remaining in its original form up to 100 min after its administration. In contrast, at P4, 30 min after the drug administration, only 55% remained as paracetamol, while paracetamol-glucuronide and paracetamol-sulfate made up 20% and 25% respectively. In the adults, the proportion of paracetamol conjugated metabolites increased further with 24% detected as paracetamol-glucuronide and 29% as paracetamol-sulfate, while only 37% remained as paracetamol itself (Fig. 11). In the rat, activity of sulfotransferases and UDP-glucuronyl transferases are limited during development and increase with age [23, 24], consistent with observations in the present study. Paracetamol-glutathione at all ages was below the linear response range (< 0.1 µg/ml) and therefore could not be quantified, similar to observations of Flint and colleagues [22], potentially due to glutathione metabolic pathway comprising a relatively small proportion of paracetamol’s metabolism (< 10%) [25]. In contrast, in humans the contribution of glucuronidation to paracetamol metabolism increases with age while sulfation decreases [22, 26].

### Effects of combination therapies on paracetamol metabolism in plasma

Paracetamol, often taken by self-medication in the treatment of headache or chronic pain [27] when patients are on treatment for other conditions; this is a particular problem in the case of pregnant women and infants [28, 29]. One of the main mechanisms by which paracetamol pharmacokinetics may be affected by other drugs is via its metabolic pathways. In adult humans, paracetamol is primarily metabolized by phase II conjugation in the liver with 55–60% glucuronidation via UDP-glucuronosyltransferase (UGT) enzymes, 20–30% sulfation via sulfotransferase (SULT) enzymes [25]. Up to 10% turns into the hepatotoxic metabolite N-acetyl-p-benzoquinone-imine (NAPQI) via cytochrome P450 enzymes [25]. This is then detoxified via glutathione conjugation by glutathione S-transferase (GST) enzymes, around 4% is excreted unchanged in urine [25]. Another mechanism that could be affected by drug interaction is paracetamol absorption. For the clinical therapeutic dose of 15 mg/kg [1], paracetamol has a plasma half-life of around 2 h, while in neonates this is extended to around 3.5 h [26] as sulfation becomes the primary route of metabolic conversion [26]. In rats, a significantly shorter half-life of around 16 min has been reported following a similar single dose of 15 mg/kg [30].

We have investigated if co-therapies with drugs such as cimetidine, digoxin, fluvoxamine, lamotrigine, lithium, olanzapine or valproate could affect its tissue entry. Following paracetamol combination therapy with 3 out of 7 drugs tested (cimetidine, lamotrigine and olanzapine), the proportion of paracetamol and its metabolites in plasma changed in an age-dependent manner. The proportion of glucuronide conjugation in paracetamol monotherapy was only 3% at E19, however cimetidine, lamotrigine and olanzapine co-therapy appeared to increase this process to 20%, 32% and 23% respectively. At P4, both lamotrigine and olanzapine co-therapy increased proportion of paracetamol-glucuronide compared to paracetamol monotherapy while decreasing the proportion of paracetamol-sulfate. In adults, olanzapine co-therapy appeared to decrease paracetamol-glucuronide and increase paracetamol-sulfate production (Fig. 11B-D).

### Brain and CSF entry of paracetamol and metabolites in monotherapy

Parent paracetamol entry into the brain remained at just over 100% throughout development while into the CSF was around 100% in the younger animals only, decreasing to around 70% in adults (Fig. 3). This observed decreased CSF entry could be due to developmental increase of CSF flow and turnover (sink effect [31, 32]) and/or increasing overall size of the ventricular system and resulting volume of distribution [33]. When the concentrations of metabolites (paracetamol-glucuronide and paracetamol-sulfate) were added together to that of paracetamol (Fig. 11) the resulting calculated apparent brain and CSF entry of paracetamol declined with age, similar to previously published data [7].

### Effects of combination therapy

In combination therapy, paracetamol brain and CSF entry was challenged using 7 drugs (cimetidine, digoxin, fluvoxamine, lamotrigine, lithium, olanzapine, valproate). Some of these may be taken in combination e.g. valproate and lamotrigine in epilepsy [34] or with paracetamol which may be self-medicated for headache or other painful conditions [29]. This part of the study was designed to investigate the potential of pharmacological modulation of paracetamol cellular transfer by affecting responsible efflux and influx mechanisms [13, 16] in addition to its metabolism.

It is known that several ABC efflux transporters such as MRPs (particularly ABCC2, ABCC3 and ABCC4) as well as BCRP (ABCG2) [35, 36], reviewed by Mazaleuskaya and colleagues [25], can transport paracetamol metabolites. Cimetidine has been described as both a substrate for BCRP and an inhibitor of many cytochrome P450 enzymes [37, 38]. These enzymes are also responsible for the formation of toxic paracetamol metabolite NAPQI [25]. However, studies investigating interactions between paracetamol and cimetidine found that when the two were co-administered in adults, there were no significant effects on paracetamol pharmacokinetics [39, 40]. This partially aligns with the results from the present study where concentrations of paracetamol metabolites in adults did not seem to be affected by cimetidine co-administration (Fig. 6). In contrast, in younger animals following cimetidine co-therapy, paracetamol-sulfate concentration significantly decreased at E19 (Fig. 4). Previous studies have investigated the ability of fetuses to metabolise drugs and have reported that gene expression and protein distribution of several cytochrome P450 enzymes were negligible in the human fetus (reviewed by Lindemalm and van den Anker [41]). These enzymes were also not present in any of the rat RNA-sequencing datasets (brain cortex, choroid plexus and placenta at E19) obtained in our previous study [42], suggesting that changes induced by cimetidine at E19 could be due to interactions with ABC transporters such as BCRP rather than inhibition of metabolising enzymes.

Another change demonstrated in the present study was increased placental transfer of paracetamol-sulfate when lamotrigine was co-administered (Fig. 9). In the brain, lamotrigine has been reported to be transported by influx transporter OCT1 (SLC22A1) [43] and efflux transporters BCRP and Pgp [44, 45]. As SLC22A1 is an influx transporter, it may be expected that an increase in transporter expression is accompanied by an increase in drug entry, whereas the opposite would be true for the efflux transporters BCRP and Pgp. However, this does not appear to be the case in the placenta in a study that showed that instead, an unidentified carrier is likely involved in lamotrigine intake into placental cells [46]. Previous work has also found that when paracetamol and lamotrigine are co-administered, the half-life of lamotrigine decreased and its clearance increased [47, 48]. The mechanisms involved in this induction are thought to be due to interactions between glucuronidation enzymes [48]. Both paracetamol and lamotrigine are substrates for several glucuronosyltransferases [25, 49, 50]. Potential interactions between these enzymes could provide a plausible explanation for the increase in paracetamol transfer when combined with lamotrigine.

In the present study, drug entry into the brain was expressed as a ratio between its concentrations in the brain over that in plasma. Rather than an increase in paracetamol concentration in the brain, the observed apparent increase in this ratio was in fact due to its decreased concentration in plasma (Supplementary Table S1). Correspondingly, the observed decrease in paracetamol transfer into fetal CSF may be due to the decrease in its transfer across the placenta rather than action at the fetal barriers themselves (Fig. 10). Similar to cimetidine, paracetamol and olanzapine also share cytochrome P450 enzymes, CYP1A2 and CYP2D6 in their metabolism pathways. In the case of olanzapine, they facilitate the formation of N-demethyl olanzapine or 2- hydroxymethyl olanzapine (Korprasertthaworn et al., 2015) respectively. However, this does not explain the effects of olanzapine on paracetamol concentration and its transfer. At E19 concentration of only paracetamol itself changed and not of paracetamol-glucuronide or paracetamol-sulfate, hence changes due to interactions between UGT or SULT enzymes are unlikely. Instead, this decrease in paracetamol concentration in plasma could be due to action at an ABC efflux transporter, resulting in decreased efflux of paracetamol. Olanzapine is known to inhibit the activity of several ABC efflux transporters including Pgp and MRP5 [51], these transporters in particular have previously been suggested to contribute to paracetamol excretion [52]. An alternative explanation may be that olanzapine facilitates an increase in an alternate metabolism pathway such as via transient receptor potential vanilloid type 1 (TRPV1) agonist N-arachidonoylphenolamine (AM404 via FAAH). The functional effect of olanzapine co-treatment on paracetamol transfer does not appear to be established, however, the effect of paracetamol on olanzapine transfer in a reverse experiment has been published previously [13]. In that study, paracetamol co-administration also decreased olanzapine transfer across the placenta.

#### Clinical implications

There are well-known differences in the metabolism of paracetamol in humans and rats, but results from the present study bring to attention that the amount of this drug that enters the brain may be dictated by its metabolism by the liver rather than by the cellular control by efflux/influx transporters at the brain barriers. Compared to younger ages, adults would need to take more paracetamol to get the desired analgesic/ antipyretic effect due to the higher rate of its metabolism. It is also not known if these metabolites can elicit similar pharmacological effects. In addition, other drugs appear to interfere with paracetamol metabolism, changing the fraction of water-soluble paracetamol metabolites that are restricted from entering the brain by blood-brain barrier interfaces.

### Limitations

High doses of paracetamol are known to be toxic due to the accumulation of N-acetyl-p-benzoquinone imine (NAPQI). However, the dose given in the present study was well within the clinically recommended range and coupled with placental drug restriction the concentration of NAPQI especially in the fetus was unlikely to have reached toxic levels.

As it is always preferable to use animals from several litters, every effort was made to include individuals from at least two litters. However, in drug combination studies, due to ethical limitations, in most cases only one dam was used for each treatment group, resulting in 6 dams in total. Therefore, the changes observed have to be interpreted with caution. Nevertheless, they do appear to line up well with previously reported values [13].

Finally, only a single dose of paracetamol was applied in the present study as the aim was to look at the interference between the drugs in an acute setting rather than changes in cellular processes in prolonged exposure. However, it would be interesting to investigate if long-term paracetamol exposure in combination therapies changes its transfer across the placenta and into the brain, which could be due to potential changes in metabolising enzymes and efflux or influx transporters.

## Electronic supplementary material

Below is the link to the electronic supplementary material.

Supplementary Material 1

Supplementary Material 2

## Data Availability

No datasets were generated or analysed during the current study.

## Abbreviations

ABC: ATP-binding cassette
BCRP: Breast cancer resistance protein
CIM: Cimetidine
CSF: Cerebrospinal fluid
DIG: Digoxin
E: Embryonic day
FLX: Fluvoxamine
GST: Glutathione S-transferase
LC: MS-Liquid chromatography coupled with mass spectrometry
Li: Lithium
LTG: Lamotrigine
NAPQI: N-acetyl-p-benzoquinone-imine
OLZ: Olanzapine
P: Postnatal day
PARA: Paracetamol
Pgp: P-glycoprotein
SLC: Solute carrier
SULT: Sulfotransferase
UGT: UDP-glucuronosyltransferase
VPA: Valproate
