# Supplementary material for: Paracetamol, its metabolites, and their transfer between maternal circulation and fetal brain in mono- and combination therapies
Source: Pharmacol Rep. 2025 Jan 24;77(2):474–89. doi: 10.1007/s43440-024-00682-6 (PMC11911254; doi:10.1007/s43440-024-00682-6)
Supplement: Supplementary file 1 — Supplementary Material 1 [file 43440_2024_682_MOESM1_ESM.pdf]

Supplementary Figure

Title: Paracetamol, its metabolites, and their transfer between maternal circulation and fetal brain in mono- and combination therapies

Journal: Pharmacological Reports

Authors: Yifan Huang, Fiona Qiu, Katarzyna M Dziegielewska, Mark D Habgood, Norman R Saunders

Corresponding author: Yifan Huang (yifan.huang1@monash.edu), Department of Neuroscience, Monash University, Melbourne Victoria, 3004, Australia

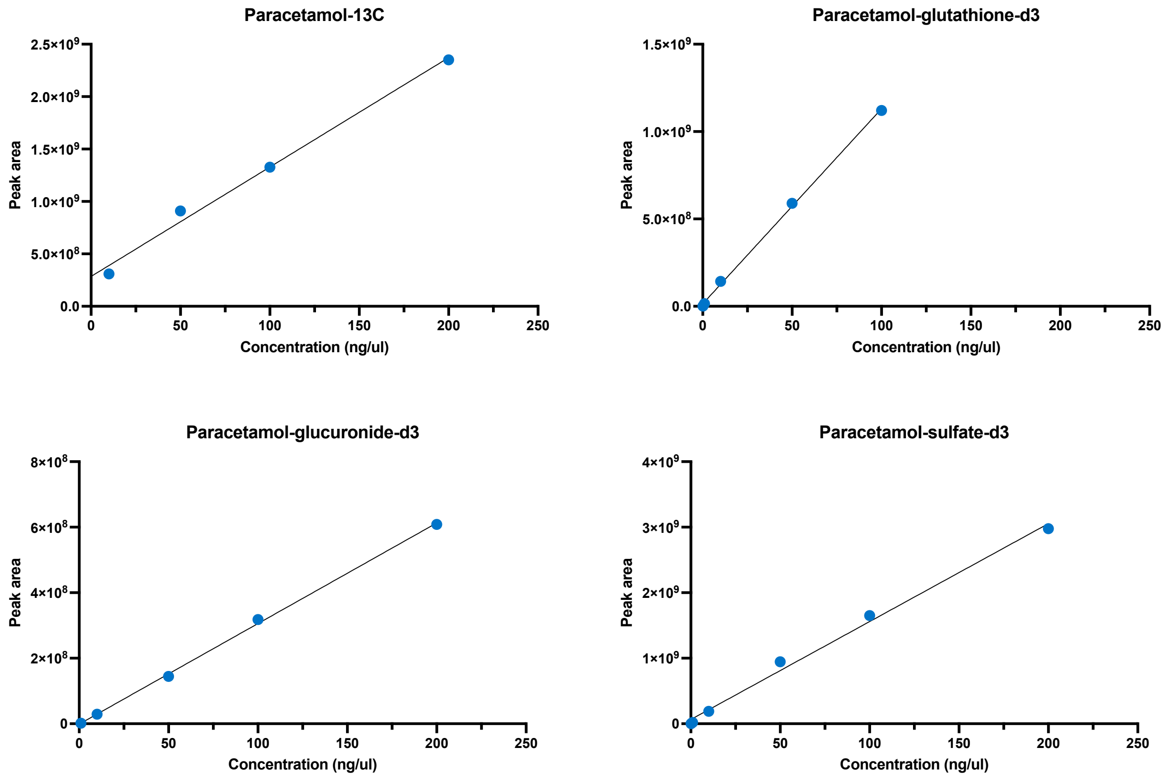

Supplementary Figure S1. Standard curves of paracetamol (**A**) 13C, (**B**) glutathione-d3, (**C**) glucuronide-d3 and (**D**) sulfate-d3 measured using liquid chromatography coupled with mass spectrometry (LC-MS).
