# Supplementary material for: Paracetamol, its metabolites, and their transfer between maternal circulation and fetal brain in mono- and combination therapies
Source: Pharmacol Rep. 2025 Jan 24;77(2):474–89. doi: 10.1007/s43440-024-00682-6 (PMC11911254; doi:10.1007/s43440-024-00682-6)
Supplement: Supplementary file 2 — Supplementary Material 2 [file 43440_2024_682_MOESM2_ESM.pdf]

Supplementary Tables

Title: Paracetamol, its metabolites, and their transfer between maternal circulation and fetal brain in mono- and combination therapies

Journal: Pharmacological Reports

Authors: Yifan Huang, Fiona Qiu, Katarzyna M Dziegielewska, Mark D Habgood, Norman R Saunders

Corresponding author: Yifan Huang (yifan.huang1@monash.edu), Department of Neuroscience, Monash University, Melbourne Victoria, 3004, Australia

Treatment	Sample	μg/ml			
		Paracetamol	Paracetamol-Glutathione	Paracetamol-Glucuronide	Paracetamol-Sulfate
PARA n=10	Brain	9.3 (5.0)	0.01	N.D.	0.04 (0.03)
	CSF	8.0 (3.2)	N.D	N.D.	0.09 (0.05)
	Plasma	7.5 (2.9)	0.01 (0.02)	3.81	1.5 (0.4)
PARA+CIM n=10	Brain	10.2 (5.7)	N.D.	N.D.	0.02 (0.02)
	CSF	7.2 (1.7)	N.D.	N.D.	0.06 (0.04)
	Plasma	7.6 (2.5)	N.D.	3 (1.5)	1 (0.2)
PARA+DIG n=9	Brain	6.6 (3.0)	N.D.	0.5	0.01 (0.01)
	CSF	6.5 (2.2)	N.D.	0.2	0.05 (0.04)
	Plasma	5.8 (2.2)	0.04	3.9 (4.2)	0.9 (0.4)
PARA+LTG n=10	Brain	8 (2.6)	N.D.	N.D.	0.02 (0.02)
	CSF	6.3 (3.4)	N.D.	N.D.	0.08 (0.05)
	Plasma	6.2 (3.7)	0.01 (0.003)	3.9 (0.9)	1.1 (0.4)
PARA+OLZ n=10	Brain	8.7 (3.9)	N.D.	0.7	0.05 (0.03)
	CSF	4.7 (4.0)	N.D.	0.3, 1	0.09 (0.06)
	Plasma	4.1 (3.7)	N.D.	3.0 (2.4)	1.4 (0.5)
PARA+VPA n=10	Brain	6.9 (2.1)	0.01	N.D.	0.01, 0.03
	CSF	5.9 (2.1)	N.D.	N.D.	0.06 (0.03)
	Plasma	6.0 (2.5)	0.01 (0.01)	2.4, 1.1	0.9 (0.3)

Supplementary Table S1. Concentration (μg/ml) of paracetamol and its metabolites (glutathione-, glucuronide- and sulfate- conjugated paracetamol) in embryonic day 19 (E19) rats following acute maternal treatment of paracetamol (15 mg/kg) in mono- or combination therapy measured using LC-MS. Values are median (IQR). For groups with less than 3 detected samples, all values are listed. N.D. (not detected) are values <0.01μg/ml. One pregnant dam was used for each treatment group. Cimetidine (CIM), digoxin (DIG), lamotrigine (LTG), olanzapine (OLZ), paracetamol (PARA), valproate (VPA).

Treatment	Sample	µg/ml			
		Paracetamol	Paracetamol-Glutathione	Paracetamol-Glucuronide	Paracetamol-Sulfate
PARA n=4	Brain	8.9 (2.1)	N.D.	N.D.	0.09 (0.03)
	CSF	7.0 (2.8)	N.D.	0.2 (0.1)	0.09 (0.05)
	Plasma	5.6 (0.7)	0.03 (0.01)	4.4 (1.7)	3.1 (1.8)
PARA+CIM n=4	Brain	9.0 (1.3)	N.D.	N.D.	0.06 (0.02)
	CSF	6.2 (2.1)	N.D.	0.2 (0.04)	0.1 (0.02)
	Plasma	10.6 (10.3)	0.01 (0.04)	7.8 (2.5)	7.5 (1.7)
PARA+DIG n=4	Brain	10.3 (5.7)	N.D.	0.1	0.07 (0.04)
	CSF	6.3 (2.9)	N.D.	0.1 (0.03)	0.1 (0.02)
	Plasma	8.2 (1.1)	0.02 (0.01)	6.6 (1.4)	7.6 (3.3)
PARA+FLX n=4	Brain	8.7 (1.9)	N.D.	0.1	0.04, 0.05
	CSF	9.4 (6.5)	0.04	0.2 (2)	0.1 (1.6)
	Plasma	6.5 (6.1)	0.05	6.5 (1.2)	5.7 (1.4)
PARA+LTG n=5	Brain	9.9 (1.7)	0.03 (0.01)	N.D.	0.06 (0.02)
	CSF	7.2 (1.3)	N.D.	N.D.	0.07 (0.02)
	Plasma	7.2 (3)	N.D.	10.1 (8.0)	3.8 (0.9)
PARA+Li n=4	Brain	7.6 (4.9)	N.D.	0.2	0.08 (0.01)
	CSF	4.7 (2.0)	N.D.	0.1 (0.05)	0.1 (0.04)
	Plasma	4.3 (4.4)	0.02 (0.03)	5.8 (2.2)	7.2 (1.2)
PARA+OLZ n=4	Brain	6.9 (2.1)	0.05	N.D.	0.02
	CSF	5.3 (1.9)	N.D.	0.5	0.05±0.02
	Plasma	5.6 (1.9)	0.01 (0.01)	10.7 (6.4)	2.7 (1.0)
PARA+VPA n=4	Brain	5.3 (3.1)	0.03 (0.01)	N.D.	0.05, 0.05
	CSF	6.4 (1.0)	N.D.	N.D.	0.06 (0.01)
	Plasma	6.7 (3.9)	0.02, 0.01	6.6 (11.4)	4.1 (2.1)

Supplementary Table S2. Concentration (µg/ml) of paracetamol and its metabolites (glutathione-, glucuronide- and sulfate- conjugated paracetamol) in postnatal day 4 (P4) rats following acute treatment of paracetamol (15 mg/kg) in mono- or combination therapy measured using LC-MS. Values are median (IQR). For groups with

less than 3 samples, individual values are listed. N.D. (not detected) are values <0.01μg/ml. Two litters were used in each treatment group, except in paracetamol monotherapy where one litter was used. Cimetidine (CIM), digoxin (DIG), fluvoxamine (FLX), lamotrigine (LTG), lithium (Li), olanzapine (OLZ), paracetamol (PARA), valproate (VPA).

Treatment	Sample	µg/ml			
		Paracetamol	Paracetamol-Glutathione	Paracetamol-Glucuronide	Paracetamol-Sulfate
PARA n=6	Brain	8.1 (1.2)	0.1	2.0	0.1 (0.1)
	CSF	5.3 (3.3)	N.D.	N.D.	0.02 (0.01)
	Plasma	8.4 (1.9)	0.05 (0.04)	10.8 (11.7)	8.8 (5.8)
PARA+CIM n=3	Brain	2.9 (2.4)	N.D	N.D.	0.2
	CSF	2.1 (1.1)	N.D.	N.D.	0.01
	Plasma	3.4 (1.0)	0.02, 0.02	7, 15	5.5 (1.4)
PARA+DIG n=3	Brain	4.3 (1.6)	0.01	N.D.	0.02, 0.02
	CSF	4.2 (1.4)	N.D.	N.D.	0.02
	Plasma	4.4 (1.6)	0.03 (0.01)	22.4 (18.7)	7.2 (2.2)
PARA+FLX n=4	Brain	4.8 (0.6)	0.1	0.2, 1	0.1 (0.4)
	CSF	4.4 (0.3)	N.D.	0.05 (0.04)	0.07 (0.03)
	Plasma	4.6 (1.0)	0.08 (0.03)	8.6 (3.7)	12.9 (1.0)
PARA+LTG n=3	Brain	7.1 (0.7)	N.D.	N.D.	0.1, 0.1
	CSF	4.8 (0.3)	N.D.	N.D.	0.02, 0.04
	Plasma	7.5 (1.0)	0.01, 0.004	19.3 (2.5)	11.5 (0.7)
PARA+OLZ n=4	Brain	3.9 (1.6)	N.D.	0.1	0.2 (0.2)
	CSF	4.2 (1.7)	N.D.	0.1	0.05 (0.5
	Plasma	6.7 (2.7)	0.07 (0.03)	4.5 (1.1)	14.8 (2.7)
PARA+VPA n=3	Brain	7.5 (3.0)	N.D.	N.D.	0.1
	CSF	4.7 (0.5)	N.D.	N.D.	0.01
	Plasma	7.5 (1.3)	0.07 (0.05)	8.8, 11.2	12.1 (0.4)

Supplementary Table S3. Concentration (µg/ml) of paracetamol and its metabolites (glutathione-, glucuronide- and sulfate- conjugated paracetamol) in non-pregnant adult rats following acute treatment of paracetamol (15 mg/kg) in mono- or combination therapy measured using LC-MS. Values are median (IQR). For groups with less than 3 samples, individual values are listed. N.D. (not detected) are values <0.01µg/ml. Cimetidine (CIM), digoxin (DIG), fluvoxamine (FLX), lamotrigine (LTG), olanzapine (OLZ), paracetamol (PARA), valproate (VPA).
